# Trauma Evaluation and Management TEAM® course for medical students in Pakistan

**DOI:** 10.12669/pjms.36.6.2588

**Published:** 2020

**Authors:** Rufina Soomro, Sobia Ali

**Affiliations:** 1 Dr. Rufina Soomro, MBBS, FCPS. Department of General Surgery, Liaquat National Hospital & Medical College, Karachi, Pakistan; 2 Dr. Sobia Ali, MBBS, MHPE. Department of Health Professions Education, Liaquat National Hospital & Medical College, Karachi, Pakistan

**Keywords:** Trauma, Undergraduate medical curriculum trauma teaching, developing countries, TEAM

## Abstract

**Objectives:**

To assess the immediate effect of TEAM® on trauma related knowledge of undergraduate medical students and to highlight the stakeholders’ acceptability of TEAM® for trauma training of undergraduate medical students

**Methods:**

Effectiveness of TEAM® course in terms of knowledge gain was assessed using 20-item-MCQs at three different timings to three cohorts of medical students from year 2017 (Group A), 2018 (Group B) and 2019 (Group C). Group A attempted the test after traditional teaching in wards, Group B attempted it after reading books and videos of TEAM®, along with traditional trauma teaching. Finally Group C attempted the test after TEAM® course along with videos and books. Students and faculty also filled evaluation questionnaire for their acceptability assessment. Kruskal-Wallis Test was applied for comparison between scores of the three groups. The evaluation questionnaire of students as well as for faculty was evaluated by determining frequencies and percentages.

**Results:**

A statistically significant difference is found after comparing the scores of the three groups (p< 0.00). More than 85% of the students were of a view that this course would help in their future practice and application. Similarly, 80% of the faculty would prefer to be involved in TEAM® teaching in future.

**Conclusion:**

There is an improvement in trauma cognitive knowledge, after the TEAM® program. Students and faculty strongly supported its introduction in the undergraduate curriculum and hence acceptable to both.

## INTRODUCTION

Despite the fact that the first line management of severe trauma cases are done by junior doctors^1^, trauma evaluation and management skills are still relatively less taught in medical school undergraduate curriculums worldwide. Several authors have drawn attention to the insufficient trauma training in medical schools, but little work has been done to address this neglected area in undergraduate curriculum.^2^,^3^ A study from the UK also reported that students emphasized on the lack of proper and adequate trauma training during their medical school.^1^

As a solution to this problem, ATLS developed by the American College of Surgeons has been introduced in some medical colleges of developed countries.^4^ Studies showed that teaching ATLS to medical students leads to significant increase in knowledge and skill, and is well received.^5^-^7^ However, high course fee due to the royalty payable to the American College of Surgeons, trained faculty requirement, and high resource requirement are major obstacles to ATLS teaching at undergraduate level in developing countries.^8^ In addition, experts are of the view that skills taught in ATLS are quite advance for the undergraduate medical students’ level.^9^ To address the identified issues of ATLS training for medical students, American College of Surgeons Committee on Trauma (ACS COT) designed Trauma Evaluation and Management (TEAM®) for senior medical students. TEAM® program, a shorter version of ATLS intended as an introduction to trauma care for medical students. It has been implemented for trauma teaching to medical students in various developed and developing countries and is shown to improve trauma education.^2^,^3^,^10^-^13^ Literature from Pakistan is mainly focused on service components of trauma, with a dearth of studies exploring the need and implementation of a structured trauma course in existing medical school curriculum in general and on TEAM® implementation or assessment in particular.^14^-^16^ Riaz et al in their literature review stated that undergraduate medical students are exposed to trauma patients during their surgical clerkships, but there is no structured or formal curriculum of trauma training in Pakistan.^17^ Keeping the need of formal trauma training in view, Liaquat National Hospital & Medical College (LNH&MC) decided to introduce TEAM® to its fourth year MBBS students in their Orthopedic and Trauma Module.

### Rationale and objectives

Because using TEAM® as a primary instructional strategy for undergraduate trauma teaching is relatively a new development in Pakistan, its role on change of knowledge regarding trauma is required to be assessed. Similarly, acceptability by facilitators and students regarding the strategy for its continuous practice and implementation in our institute, as well other institutes of our country, need to be addressed. This study will assess the immediate effect of TEAM® on trauma related knowledge of undergraduate medical students. It will also highlight the methodology of implementation and stakeholders’ acceptability of TEAM® for trauma training of undergraduate medical students.

## METHODS

The placement of this course is aligned with the affiliated University Orthopedic and Trauma Module for 4^th^ year MBBS students. This course was offered to all students for the year 2017, 2018, and 2019. This study was approved by the Ethical Committee (Ref: App # 0509- 2020- LNH-ERC, Dated: March 4, 2020) of Liaquat National Hospital and Medical College, Karachi.

The five hour TEAM® course was taught to three cohorts of medical students from semester VII in 2017, 2018 and 2019. The course was conducted on four consecutive Thursdays (25 students in each batch for each single cohort) with a 45 minute lunch break in between. A multidisciplinary faculty (that included ATLS instructors as Course Directors and, ATLS certified individuals) were invited to conduct the sessions. After explaining the objectives, the students were shown a video in which a doctor commits multiple critical errors in the assessment and management of a trauma patient. This was then followed by a lecture adopted from the TEAM® program highlighting the appropriate diagnostic and resuscitative measures. At the end of this lecture, another video was shown in which most of the errors of the first video were corrected. After the video, there was a demonstration of instruments used for trauma patient management. A break was then offered for lunch, which was followed by six stations. Out of six, three stations were focused on skills training; including Application of Cervical Collar & Helmet Removal, Log roll, removal of spinal board & application of pelvic binder and a separate station on how to apply traction splint. Two stations dealt with focused discussion on Poly-trauma patient scenario and Disaster management. The sixth station was a scenario based management of a trauma victim. It had a trained simulated moulaged patient. The students were expected to simulate a systematic assessment and management of that patient.

**Fig.1:**
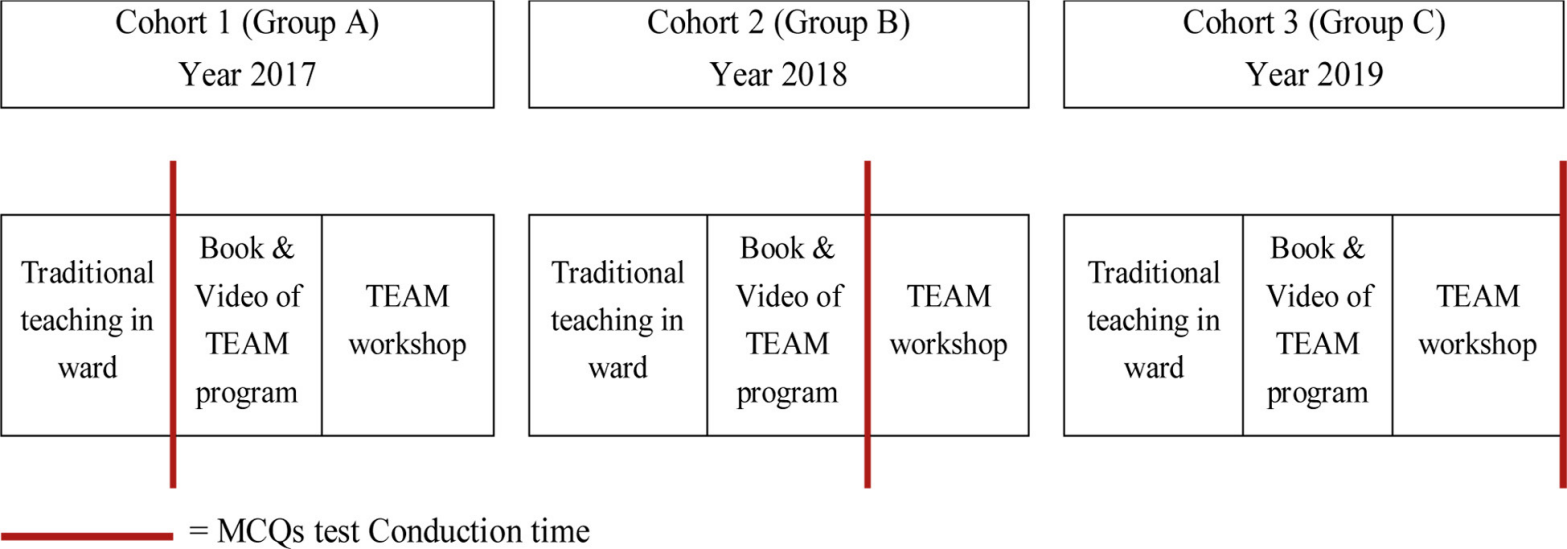
MCQs test conduction sequence.

Students were informed that the effectiveness of this program in terms of knowledge gain will be assessed by conducting a 20 item MCQs tests. This test was prepared and sent by the American College of surgeons, TEAM® course developers. The students were also notified that the results of this test will have no impact on their internal assessment scores. In order to assess effectiveness of TEAM® course the same test was conducted at three different timings to three cohort of students during the Trauma Module (Fig.1). For the cohort of 2017 (Group A), students were asked to attempt the MCQs test after traditional teaching in wards. For the year 2018 (Group B), along with traditional trauma teaching, books and videos of TEAM® were given to students and then they were given the same test for their knowledge assessment. Finally for the cohort of 2019 (Group C), along with videos and books, students had structured and standardized TEAM® program and then they were assessed by the same MCQs test. For the first two cohorts i.e. Group A & B, the structured TEAM® was introduced after the test conducted on the same standardized structure, so that no cohort can be left untrained. In addition to MCQs test, feedback from the faculty as well as from the students was taken by an evaluation questionnaire.

After completing the training, students from all three groups completed an evaluation questionnaire. This questionnaire consisted of items about lecture content, video demonstrations, skill and focused discussion stations. They were graded on a scale of 1-5 where 1 being the lowest grade and 5 being the highest. Comments were also asked about the stations. At the end, students were asked their views about the time and practicality of the program. Faculty was also invited to give their feedback through Google form. These forms were sent to the faculty immediately after training.

### Data Analysis

MCQs test scores of the three cohorts were compared after normality testing using Shapiro-Wilk’s test that showed non-normal distribution. Kruskal-Wallis Test was applied for comparison between scores of three groups. To find the difference among the groups’ score with TEAM® and those without TEAM®, Mann-Whitney test was used as a post-hoc.

The results of the evaluation questionnaire of students for the year 2018 (Group B) and year 2019 (Group C) were analyzed according to the percentage of response in each category in the feedback form. Similarly, acceptability by faculty (for the same years) was done by determining frequencies and percentages.

## RESULTS

The score comparison between the three groups using different modalities for trauma teaching are summarized in Table-I. A statistically significant difference is found between the scores of the three groups (p< 0.00)

**Table-I:** Test scores comparison among three groups (using Kruskal-Wallis).

Group	N	Median	P-Value
Group A (cohort of 2017)	105	6.00	0.000*
Group B (cohort of 2018)	92	8.00	
Group C (cohort of 2019)	97	9.00	

The results of Mann-Whitney that elaborates the difference in scores among those who attempt test after TEAM® training and those who did before the training are shown in Table-II. A statistically significant difference is found in this comparison (p=0.000).

**Table-II:** Assessment scores comparison of groups with and without TEAM teaching (using Mann-Whitney).

	Median score in MCQs	p- value
Groups without TEAM teaching ( Group A+B)	7	0.00
Group with TEAM teaching ( Group C)	9	

### Evaluation questionnaires

Students’ feedback: Table-III showed that on the scale of 1-5 with 5 being the highest, the percent of students’ assigning the respective rating for the group B & C.

**Table-III:** Students’ feedback.

	1		2		3		4		5	

	Gp B	Gp C	Gp B	Gp C	Gp B	Gp C	Gp B	Gp C	Gp B	Gp C
Course content was consistent with the stated objectives	0	0	0	0	10.1	6.2	0	0	89.9	93.8
Course content was relevant to my educational needs	0	0	0	0	6.7	10.3	0	0	93.3	89.7
Lecture explains basic concepts of trauma evaluation and management clearly	0	0	1.1	0	13.5	8.2	0	0	85.4	91.8
Initial assessment demonstration videos were engaging and relevant to the course content	0	0	3.4	1	23.6	23.7	3.4	8.2	69.7	67
The content was organized and easy to follow in skill stations	0	0	0	0	2.2	1	15.7	16.5	82	82.5
Discussion time was adequate and enhanced my understanding of the subject	0	0	1.1	0	7.9	10.3	0	0	91	89.7
Discussion sessions’ speakers were informative and knowledgeable	0	0	0	0	12.4	9.3	11.2	13.4	76.4	77.3
Experience with simulated patient will improve my performance in actual clinical setting	0	0	2.2	1	15.8	10.3	0	0	82	88.7
Acquired knowledge will be applied to my practice environment	0	0	1.1	0	11.2	3.1	0	0	87.7	96.9

Faculty feedback: Table-IV shows that on the scale of 1-5 with 5 being the highest, the percent of faculty assigning the respective ratings for the group B & C

**Table-IV:** Faculty feedback.

	Group B		Group C	

	Mean	Percentage	Mean	Percentage
The TEAM course had meaningful content	4.55	91%	4.57	91%
The Course is well placed within the curriculum	4.09	82%	4.43	89%
I found the handouts, videos and other reading material helpful and relevant	4.45	89%	4.36	87%
I found the TEAM organizers overall helpful in guiding me through the course	4.55	91%	4.71	94%
I found the TEAM organizers helpful in guiding the students through the course	4.73	95%	4.71	94%
My queries were clarified appropriately before the start of the course	4.27	85%	3.93	79%
I was given appropriate training support to conduct my session	4.45	89%	3.79	76%
I was able to engage positively with the students during the session	4.55	91%	4.57	91%
I can confidently conduct future TEAM courses	4.64	93%	4.64	93%
Effective use of mannequins and simulated patients and instrument display was done	4.45	89%	4.36	87%
Objectives were congruent with the learning needs of the participants	4.45	89%	4.57	91%
The pace of information provided was adequate	4.36	87%	4.50	90%
I would like to involve in this process again in future	4.36	87%	4.06	80%

## DISCUSSION

With this study, we presented the implementation of TEAM® in Pakistan for the students of 4^th^ year MBBS. The involvement of a multidisciplinary ATLS certified faculty helped us to follow the standard protocol for TEAM® teaching and assessment.

Our study established the effectiveness of the course in terms of knowledge assessment. MCQs test scores showed that TEAM® course has improved short term knowledge retention. By comparing the median scores of MCQs test of those who attended the course with those who didn’t, we provided the evidence of the course effectiveness. This immediate effect of the TEAM® course on trauma related knowledge is in line with various studies from developed as well as developing countries.^9^,^11^,^12^,^18^,^19^ Lum SK and Subramaniam T^10^ claimed that the competency of managing trauma patients is not related to students’ learning through surgical posting. They further clarified that surgical posting on topics unrelated to trauma may dilute the learning related to the trauma only. Median scores comparison between those attended TEAM® and those who didn’t, irrespective of their surgical posting (Table-I and II) also seconded this claim.

The reason for concern at this point is that even though the median score of group C is higher in comparison, the score of 9 out of 20 is low by any standard. Post-hoc analysis of the test showed reliability coefficient Cronbach’s alpha of 0.52. 3 out of 20 questions had options, which were not good distractors and were not opted by single student. We also feel that 20 questions is a small number and may not give us an adequate content reliability. We feel that increasing the number of questions to 40 like in ATLS may increase our reliability and may give us the true picture of students’ learning.

Stakeholders’ acceptability in terms of students and faculty appreciation is also evident by our study. More than 85% of the students in both groups were of a view that this course would help in their future practice and application. The higher percentage were agreed with the objectives achievement, course content relevancy with objectives, and the positive effect of discussion skills stations on their learning (> 85%). These findings are in line with previous studies that also showed the students’ appreciation of trauma training course.^9^,^13^,^18^,^20^,^21^

Our only statement that secured less agreement in students’ feedback was about video demonstration during the lecture (<70% agreement in both groups). This is contrary to previous studies that claimed that videos composed of real life examples and focused on contrasting cases, help students to attain expert-like differentiation.^22^,^23^ The probable explanation we found here is that video demonstration during the lecture may increase its duration and thus may cause boredom as compared to high level students’ engagement during skills and discussion sessions. Hence may be the reason of comparatively less scoring at this item. We are planning to address this issue by assigning a separate slot in the timetable for video demonstration before the actual training day.

High faculty ratings are also evident in our data. 80% or more showed willingness to teach this course. This is in line with previous studies that showed the faculty engagement and interest in trauma teaching.^3^,^24^ Percentages for faculty perception about training seemed comparatively less in year 2019 than in year 2018 (76% Vs. 89% for the years 2019 and 2018 respectively). The probable reason of faculty dissatisfaction may due to the fact that as we had new facilitators and we became confident in our yearly TEAM® teaching, we might have overlooked required faculty training, simply assuming that our faculty is well trained. We have now planned that we will conduct regular faculty training workshops before TEAM, in which experienced facilitator will have debriefing about the content, instructional strategies and feedback techniques. Inexperienced facilitators will be introduced to the course, and they will be paired with experienced facilitators for formal training.^24^

### Limitations of the study

Although strengthened by MCQs test scores of three years and two years’ worth of feedback data, our study has several limitations. We presented the immediate effect of trauma related knowledge by this course and didn’t discuss about long term retention of knowledge. Even though the learners were given formative feedback on their skills, we initially didn’t include the formal skills’ assessment procedures or results. Ongoing research by authors will address these limitations, in which we will assess long term knowledge retention by MCQ test and skills through Objective Structured Clinical Examination (OSCE).

## CONCLUSION

Guided by MCQs based knowledge assessment along with the stakeholders’ perception of the course, we provide the evidence that TEAM® course improves cognitive trauma knowledge and is acceptable to stakeholders. We expect that these results may help in initiating structured trauma training as part of the curriculum for senior medical students of Pakistan.

### Authors’ Contribution:

**RS:** Conceived, designed and did the editing of manuscript and responsible and accountable for the accuracy and integrity of the work.

**SA:** Did data collection, statistical analysis and manuscript writing.

**RS & SA:** Did review and final approval of manuscript.
